# Obesity-induced DNA hypermethylation of the adiponectin gene mediates insulin resistance

**DOI:** 10.1038/ncomms8585

**Published:** 2015-07-03

**Authors:** A. Young Kim, Yoon Jeong Park, Xuebo Pan, Kyung Cheul Shin, Soo-Heon Kwak, Abdulelah F. Bassas, Reem M. Sallam, Kyong Soo Park, Assim A. Alfadda, Aimin Xu, Jae Bum Kim

**Affiliations:** 1 Institute of Molecular Biology and Genetics, Seoul National University, Seoul 151-742, Korea; 2 Department of Biophysics and Chemical Biology, Seoul National University, Seoul 151-742, Korea; 3 State Key Laboratory of Pharmaceutical Biotechnology, The University of Hong Kong, Hong Kong, China; 4 School of Biological Sciences, Seoul National University, Seoul 151-742, Korea; 5 Department of Internal Medicine, Seoul National University College of Medicine, Seoul 110-799, Korea; 6 Obesity Research Center, College of Medicine, King Saud University, Riyadh 11461, Saudi Arabia; 7 Department of Molecular Medicine and Biopharmaceutical Sciences, Graduate School of Convergence Science and Technology, Seoul National University, Seoul 110-799, Korea; 8 Department of Medicine, King Saud University, Riyadh 11461, Saudi Arabia; 9 Department of Medicine, The University of Hong Kong, Hong Kong, China; 10 Department of Pharmacology and Pharmacy, The University of Hong Kong, Hong Kong, China

## Abstract

Adiponectin plays a key role in the regulation of the whole-body energy homeostasis by modulating glucose and lipid metabolism. Although obesity-induced reduction of adiponectin expression is primarily ascribed to a transcriptional regulation failure, the underlying mechanisms are largely undefined. Here we show that DNA hypermethylation of a particular region of the adiponectin promoter suppresses adiponectin expression through epigenetic control and, in turn, exacerbates metabolic diseases in obesity. Obesity-induced, pro-inflammatory cytokines promote DNMT1 expression and its enzymatic activity. Activated DNMT1 selectively methylates and stimulates compact chromatin structure in the adiponectin promoter, impeding adiponectin expression. Suppressing DNMT1 activity with a DNMT inhibitor resulted in the amelioration of obesity-induced glucose intolerance and insulin resistance in an adiponectin-dependent manner. These findings suggest a critical role of adiponectin gene epigenetic control by DNMT1 in governing energy homeostasis, implying that modulating DNMT1 activity represents a new strategy for the treatment of obesity-related diseases.

## Abstract (web summary)

The hormone adiponectin is produced by fat cells and has positive metabolic effects. Here, Kim *et al.* show that DNA methyltransferase 1 (DNMT1) represses adiponectin expression through hypermethylation of its promoter, and that inflammatory cytokines enhance DNMT1 activity in obese mice and humans.

## Graphical abstract

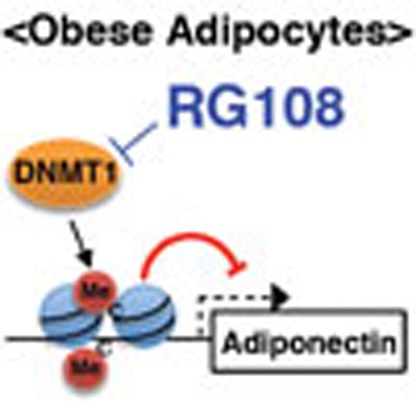

Epigenetic regulation including DNA methylation is one of the crucial mechanisms in the regulation of eukaryotic gene expression without DNA sequence modification^1^. Accumulating evidence has indicated that DNA methylation would serve as a bridge between environmental changes and cellular responses. Of note, nutrient status differentially modulates DNA methylation in several metabolic genes including hepatocyte nuclear factor 4α, pancreatic and duodenal homeobox 1 (*Pdx1*), peroxisome proliferator-activated receptor (*Ppar*) γ coactivaotr-1 α (*Pgc-1*α)^2^,^3^,^4^,^5^. Intriguingly, several reports have demonstrated that high-fat diet (HFD) consumption affects the epigenetics of various genes involved in the inheritance of metabolic imbalance to the following generation, which is associated with insulin sensitivity^6^,^7^.

Adiponectin, which is selectively expressed in adipocytes, modulates whole-body energy homeostasis by regulating glucose and lipid metabolism^8^,^9^. In metabolic tissues, adiponectin enhances insulin sensitivity via promoting glucose utilization and fatty acid oxidation^9^,^10^. In addition, adiponectin suppresses obesity-induced immune responses and development of atherogenic risk factors^11^,^12^. Accordingly, supplementation of adiponectin or adiponectin receptor agonist improves insulin sensitivity and metabolic parameters in obese animal models^13^,^14^. Interestingly, adiponectin expression and its serum levels are negatively correlated with obesity and obesity-related metabolic diseases such as insulin resistance, type 2 diabetes and cardiovascular diseases^15^.

Generally, adiponectin expression is governed in several regulatory steps such as transcriptional and post-translational regulation including oligomer assembly and secretion. In obesity, such regulatory mechanism of adiponectin is disrupted by multiple factors including adipocyte hypertrophy, inflammation and oxidative stress, leading to hypoadiponectinemia^15^,^16^,^17^. Among them, mounting evidence suggests that hypoadiponectinaemia in obesity is primarily ascribed to a failure of transcriptional regulation^15^,^18^,^19^. Importantly, the underlying molecular mechanism mediating such dysregulation in obesity has not been elucidated. In particular, it has not been explored whether epigenetic changes such as DNA methylation play roles in the dysregulation of adiponectin gene expression in obesity.

In this study, we have investigated the role of DNA methylation in adiponectin gene expression. We revealed that the hypermethylation of adiponectin promoter inhibits adiponectin transcription. Moreover, DNA methylation at the adiponectin promoter is mediated by DNA methyltransferase (DNMT) 1 whose expression is elevated in adipocytes of obese subjects. In addition, stimulation of adiponectin expression by the suppression of DNMT1 activity repressed inflammatory responses and increased insulin sensitivity in obese mice, which would provide novel insights into mechanisms underpinning the reprogramming of adiponectin gene expression by DNA methylation in metabolic complications.

## Results

### Adiponectin promoter is hypermethylated in obese subjects

Consistent with previous reports^15^,^18^,^19^, adiponectin mRNA levels were significantly reduced in adipocytes from HFD-fed obese mice, whereas mRNA levels of key adiponectin-regulatory transcription factors, including *Pparγ2* or CCAAT/enhancer-binding protein α (*C/ebp*α) were not significantly altered (Supplementary Fig. 1a). This observation led us to speculate the possibility of alternative pathways involved in the transcriptional repression of adiponectin in obesity. Among various biological processes, DNA methylation is one of the key transcriptional regulatory mechanisms governing the accessibility of the transcription machinery to target sites through modulation of the chromatin structure^20^. Furthermore, recent findings have shown that DNA methylation is an active regulatory mechanism responding to the changes in nutrient cues with multiple impacts on the regulation of systemic energy homeostasis^2^,^3^,^4^,^5^,^6^,^7^.

**Figure 1:**
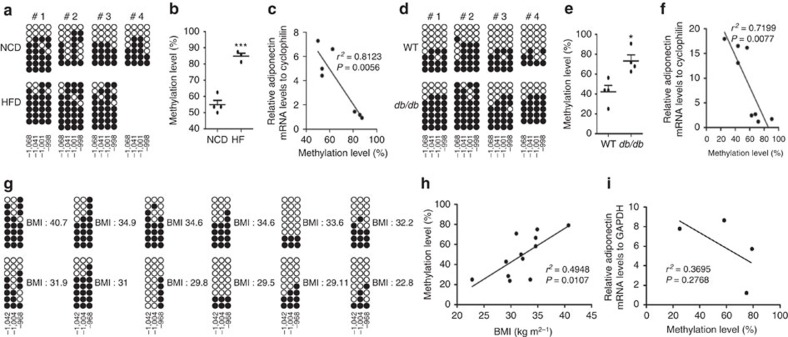
Adiponectin promoter region 2 (R2) is hypermethylated in the adipocytes of obese subjects. (**a**–**c**) R2 bisulfite sequencing analysis in adipocytes from NCD-fed (*n*=4) or HFD-fed (*n*=3) mice. (**a**) Each row indicates sequencing results of independent clones. Open circles denote unmethylated CpGs and closed circles represent methylated CpGs. The CpG position relative to upstream transcription start site of mouse adiponectin gene is shown below each column. (**b**) Percentage of R2 5-methylcytosine (5-mC). (**c**) Correlation of R2 DNA methylation and adiponectin mRNA levels. mRNA levels were measured by qPCR. *r*^*2*^ and *P* values are indicated on the graph. (**d**–**f**) R2 bisulfite sequencing results in adipocytes from WT (*n*=4) or *db/db* (*n*=4) mice. (**d**) Methylation status of each CpG. (**e**) Percentage of R2 cytosine methylation. (**f**) Correlation of R2 DNA methylation and adiponectin mRNA levels. mRNA levels were measured by qPCR. *r*^*2*^ and *P* values are indicated on the graph. (**g**–**i**) Human adiponectin promoter R2 bisulfite sequencing results in adipocytes isolated from human adipose tissue. (**g**) The CpG position relative to upstream transcription start site is shown blow each column. (**h**) Adiponectin methylation levels in human adipocytes were negatively associated with body mass index. (**i**) Correlation between adiponectin mRNA levels and R2 methylation levels in human adipocytes. mRNA levels were measured by qPCR. *r*^*2*^ and *P* values are indicated on the graph. Results are expressed as the mean±s.e.m. *P<0.05; ***P<0.001 in a two-tailed Student's *t-*test. #, individual mice.

To gain insight into the involvement of DNA methylation in the obesity-induced decrease of adiponectin expression, we used bisulfite sequencing analysis to investigate the DNA methylation levels of the adiponectin promoter in adipocytes isolated from normal chow diet (NCD)- or HFD-fed mice. There are six CpG dinucleotides within the 1.2-kb upstream region of the mouse adiponectin gene promoter (Supplementary Fig. 1b). The degree of DNA methylation of the adiponectin promoter was examined in two regions—R1 (relatively close to the transcription start site and R2 (positioned 1 kb upstream of the transcription start site; Supplementary Fig. 1b). Interestingly, the R2 was highly methylated in adipocytes from HFD-fed obese mice as compared with NCD-fed lean mice (Fig. 1a,b). Moreover, R2 methylation levels were inversely correlated with the amounts of adiponectin mRNA (Fig. 1c). Likewise, the R2 was hypermethylated and adiponectin gene expression significantly decreased in adipocytes of genetically obese *db/db* mice (Fig. 1d–f, Supplementary Fig. 1c). By contrast, the R1 methylation was unaltered regardless of obesity (Supplementary Fig. 1d–i). Notably, obesity-associated hypermethylation was specific to the adiponectin promoter, whereas methylation levels in the promoters of other genes, including *Pparγ2* or *Tnf*α, were not promptly affected (Supplementary Fig. 1j–u), suggesting that DNA methylation would participate in the obesity-induced alteration of gene expression in a gene-specific manner. Further, we examined whether human adiponectin expression was associated with DNA methylation of the human adiponectin promoter containing three CpG dinucleotides in a region that shows high homology with the R2 of the mouse adiponectin promoter (Supplementary Fig. 2). In human adipocytes, the R2 methylation was not only positively correlated with body mass index (Fig. 1g,h) but also negatively associated with adiponectin transcripts (Fig. 1i), findings that further support the potential role of DNA methylation in the R2 to mediate obesity-induced dysregulation of adiponectin expression.

### DNMT1 governs adiponectin gene expression via R2 methylation

**Figure 2:**
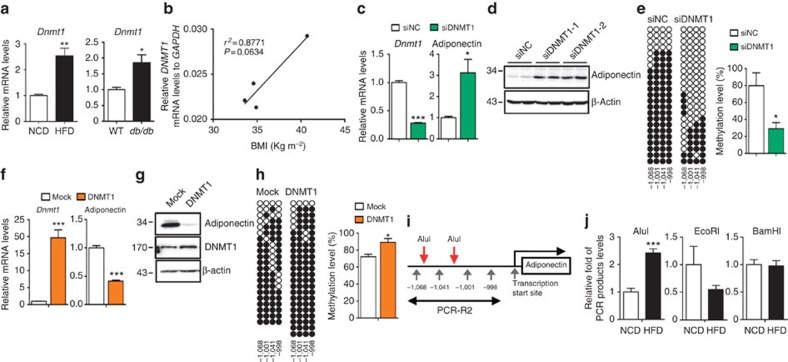
DNMT1 regulates the DNA methylation of the adiponectin promoter R2. (**a**) *Dnmt1* mRNA levels in adipocytes from NCD- (*n*=4) or HFD-fed (*n*=3) mice and in adipocytes from WT (*n*=4) or *db/db* mice (*n*=4). (**b**) Correlation between body mass index and *DNMT1* mRNA levels in human adipocytes. mRNA levels were measured by qPCR. *r*^*2*^ and *P* values are indicated on the graph. (**c**–**e**) DNMT1 was suppressed by small interfering RNA in 3T3-L1 cells (*n*=3). (**c**) *Dnmt1* and adiponectin mRNA levels. mRNA levels were measured by qPCR. (**d**) Adiponectin protein levels were determined by western blot analysis. (**e**) Bisulfite sequencing data at the R2. (**f**–**h**) DNMT1 overexpression in differentiated 3T3-L1 cells (*n*=3). (**f**,**g**) *Dnmt1* and adiponectin mRNA and protein levels were measured by qPCR and western blot analysis. (**h**) Degree of R2 DNA methylation was examined by bisulfite sequencing. (**i**) AluI restriction sites in R2. Red and grey arrows indicate the AluI restriction sites and CpG locations in the R2, respectively. Double headed arrow points to PCR amplified region. (**j**) Restriction enzyme accessibility assay in adipocytes from HFD-fed (*n*=5) or NCD-fed (*n*=5) mice. EcoRI and BamHI, which do not digest R2, were used as negative controls. Results are expressed as the mean±s.e.m. Similar results were obtained at least more than three independent experiments. *P<0.05; **P<0.01; ***P<0.001 in a two-tailed Student's *t-*test. siNC; negative control siRNA. See Supplementary Fig. 11 for full-length images of blots.

Next, we verified which DNMTs among the three isoforms in mammals, DNMT1, DNMT3a and DNMT3b, were responsible for the obesity-induced R2 hypermethylation. Examination of DNMT expression in adipocytes indicated that the level of *Dnmt1* mRNA was exclusively decremented in mature adipocytes that predominantly contribute to adiponectin expression (Supplementary Fig. 3a,b). Moreover, *Dnmt1* expression was elevated in adipocytes from HFD-fed and *db/db* mice compared with that from NCD-fed or wild-type (WT) lean mice (Fig. 2a). Importantly, *DNMT1* expression in human adipocytes showed a positive correlation with body mass index (Fig. 2b). Indeed, DNMT1 knockdown in differentiating adipocytes led to a selective increase of both adiponectin mRNA and protein expression with a significant reduction of the R2 methylation (Fig. 2c–e), whereas DNMT3a suppression did not significantly influence adiponectin gene expression (Supplementary Fig. 3c). Conversely, DNMT1 overexpression significantly decreased the adiponectin mRNA level, concomitant with hypermethylation of the R2 (Fig. 2f–h), but not the R1 (Supplementary Fig. 3d). Furthermore, mutation of all CpGs to CpCs at the R2 mitigated DNMT1-induced decrease in adiponectin promoter activity in adipocytes (Supplementary Fig. 3e), arguing that DNMT1 indeed inhibits adiponectin expression in a DNA methylation-dependent manner. Compelling evidence indicates that the suppression of gene expression by DNA methylation is linked with chromatin remodelling^20^,^21^. Notably, the two AluI restriction sites in the R2 (Fig. 2i) became resistant to AluI enzyme digestion on HFD feeding (Fig. 2j), suggesting the formation of a compact chromatin structure around the hypermethylated R2.

### Inflammatory cytokines promote DNA methylation at the R2

**Figure 3:**
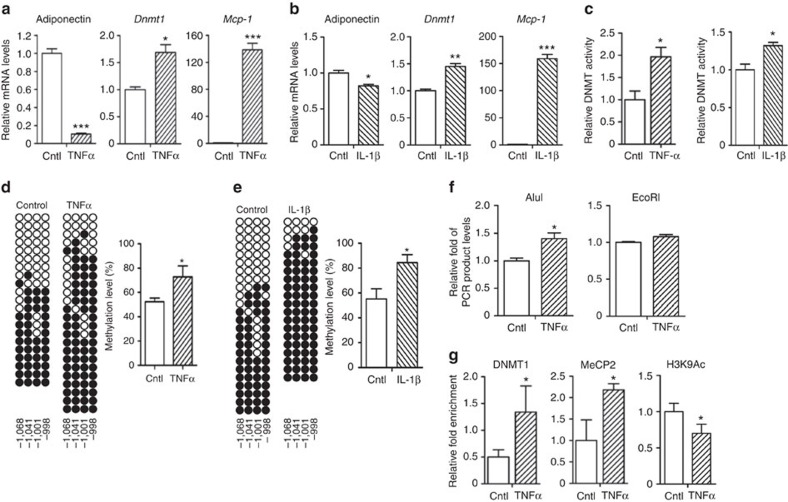
Inflammatory cytokines inhibit adiponectin expression by inducing DNMT1 expression and R2 DNA methylation. Differentiated 3T3-L1 adipocytes were incubated with or without TNFα (10 ng ml^−1^) or IL-1β (10 ng ml^−1^) for 24 h. (**a**,**b**) Adiponectin, *Dnmt1* and *Mcp-1* mRNA levels. (**c**) DNMT relative enzymatic activity. (**d**,**e**) R2 bisulfite sequencing analysis and quantification of 5-mC. (**f**) Restriction enzyme accessibility assay. mRNA levels were measured by qPCR. (**g**) R2 ChIP analysis. Results are expressed as the mean value±s.e.m. of three independent samples (*n*=3). Similar results were obtained at least more than three independent experiments. *P<0.05; **P<0.01; ***P<0.001 in a two-tailed Student's *t-*test. Cntl, control.

To understand the molecular mechanisms of obesity-induced adiponectin promoter hypermethylation, differentiated adipocytes were challenged with several stimuli suppressing adiponectin expression, including pro-inflammatory cytokines, endoplasmic reticulum stress, mitochondrial dysfunction or hypoxic environment^22^,^23^,^24^. Although the above factors potently suppressed adiponectin gene expression, only pro-inflammatory cytokines such as tumour necrosis factor (TNFα) and interleukin (IL)-1β were able to induce *Dnmt1* expression and activity (Fig. 3a,b and Supplementary Fig. 4a), potentiating hypermethylation of the R2 but not the R1 (Fig. 3c–e, Supplementary Fig. 4b, and Supplementary Fig. 5). Further, NF-κB signalling pathway appeared to be engaged in cytokine-induced stimulation of DNMT1 as treatment of Bay-11–7082 (BAY), an inhibitor of NF-κB, substantially reduced the level of DNMT1 expression induced by TNFα (Supplementary Fig. 6). In addition, TNFα induced a closed chromatin structure in the R2 (Fig. 3f) and enhanced the recruitment of DNMT1 and methyl CpG-binding protein 2 (MeCP2), a methyl-DNA-binding protein that interacts with histone-modifying enzymes to the R2 (Fig. 3g). Simultaneously, the level of H3K9 acetylation (H3K9Ac) at the R2 decreased in TNFα-treated adipocytes (Fig. 3g).

### DNMT inhibition alleviates the effect of TNFα on adiponectin

**Figure 4:**
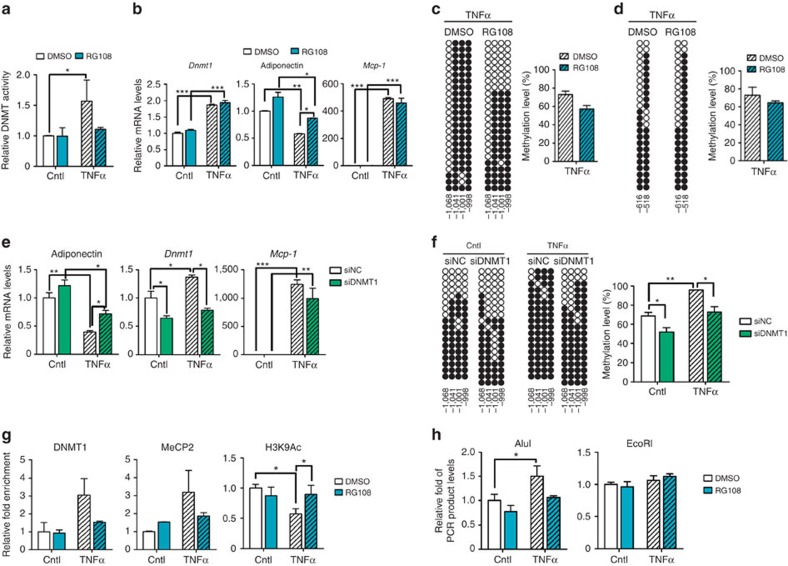
Inhibition of DNMT1 relieves TNFα-induced adiponectin gene suppression through inhibition of R2 DNA hypermethylation. (**a**–**d**) 3T3-L1 adipocytes were pretreated with DMSO (white bars) or RG108 (blue bars; 100 μM) for 24 h before TNFα treatment (hatched bars; 10 ng ml^−1^) for 24 h (*n*=3). (**a**) Relative DNMT enzymatic activity. (**b**) mRNA levels of *Dnmt1*, adiponectin and *Mcp-1*. mRNA levels were measured by qPCR. (**c**,**d**) Bisulfite sequencing results of the adiponectin promoter R2 (**c**) and R1 (**d**) in 3T3-L1 cells treated with TNFα. Quantification of the 5-mC levels in the adiponectin promoter R2 and R1. (**e**,**f**) In 3T3-L1 adipocytes, DNMT1 was suppressed by small interfering RNA. The cells were then incubated with or without TNFα (10 ng ml^−1^) for 24 h (*n*=3). (**e**) mRNA levels of adiponectin, *Dnmt1* and *Mcp-1* in negative control (NC) or DNMT1 suppressed 3T3-L1 adipocytes. mRNA levels were measured by qPCR. (**f**) R2 DNA methylation levels were measured by bisulfite sequencing. (**g**,**h**) 3T3-L1 adipocytes were pretreated with DMSO (white bars) or RG108 (blue bars; 100 μM) for 24 h before TNFα treatment (hatched bars; 10 ng ml^−1^) for 24 h (*n*=3). (**g**) R2 ChIP analysis. Quantification of DNMT1 and MeCP2 relative recruitment and H3K9Ac levels using qPCR. (**h**) Restriction enzyme accessibility assay. After restriction with endonucleases, purified gDNA was amplified and quantified using qPCR. All results are expressed as mean±s.e.m. *P<0.05; **P<0.01; ***P<0.001 in a two-tailed Student's *t*-test. Cntl, control.

To examine whether the R2 hypermethylation is involved in the inflammation-mediated downregulation of adiponectin expression, we used RG108, a DNMT inhibitor. In adipocytes, RG108 decreased TNFα-induced DNMT1 activity, while *Dnmt1* mRNA level was elevated by TNFα (Fig. 4a,b). Accordingly, RG108 attenuated the suppression of adiponectin expression by TNFα in conjunction with decrement of methylation level of the R2, but not the R1 (Fig. 4c,d). Likewise, the knockdown of DNMT1 rescued TNFα-induced the suppression of adiponectin expression with a decrease in the R2 methylation (Fig. 4e,f). RG108 also reversed TNFα-mediated decrement of H3K9Ac as well as MeCP2 recruitment and consequently augmented AluI accessibility to the R2 (Fig. 4g,h). These results strongly support the significance of DNMT1 in regulating adiponectin expression through enhancing promoter methylation and consequent alteration of chromatin structure in response to pro-inflammatory cytokines.

### DNMT inhibitor elevates adiponectin levels in *db/db* mice

**Figure 5:**
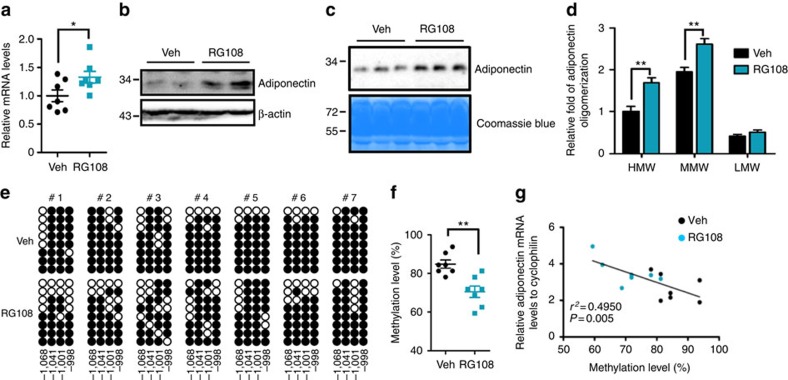
RG108 elevates adiponectin levels by the reduction of R2 DNA methylation in *db/db* mice. *db/db* mice were injected with vehicle (Veh, 1% DMSO, black circle and bar, *n*=4–7) or RG108 (blue circle, square and bar, *n*=4–7). (**a**) Adiponectin mRNA levels in adipocytes. mRNA levels were measured by qPCR. (**b**) Western blotting of adiponectin in eWAT. (**c**) Serum adiponectin levels were determined by western blot analysis. (**d**) Relative levels of each oligomeric complex of adiponectin in serum were analysed by gel filtration analysis. (**e**,**f**) R2 DNA methylation levels were examined using bisulfite sequencing in adipocytes. (**g**) Correlation between R2 DNA methylation and adiponectin mRNA levels. *r*^*2*^ and *P* values are indicated on the graph. Results are expressed as the mean±s.e.m. *P<0.05; **P<0.01 in a two-tailed Student's *t-*tests. #, individual mice. See Supplementary Fig. 11 for full-length images of blots.

Next, we administrated RG108 to *db/db* mice to explore the potential roles of DNMT1 in the dysregulation of adiponectin expression *in vivo*. In previous report, adiponectin-overexpressing *ob/ob* mice exhibit improved insulin sensitivity with elevated adiposity due to hyperplastic expansion of fat tissue^25^. Hence, we empirically determined the proper RG108 dosage (5.69 mg kg^−1^·per day) that did not affect the weight of the body or several organs (Supplementary Fig. 7a,b) and have no toxic effect on the liver function (Supplementary Fig. 7c). Conversely, RG108 significantly increased adiponectin mRNA and protein in adipose tissue, synergistically leading to the enhancement of serum adiponectin level (Fig. 5a–c). RG108 also elevated the level of high molecular weight form of adiponectin (Fig. 5d). Furthermore, the R2 methylation was significantly reduced in the adipocytes of RG108-treated *db/db* mice compared with that of vehicle-treated *db/db* mice (Fig. 5e–g). However, the R1 methylation in adipocytes and the R2 methylation in the liver were not altered by RG108 (Supplementary Fig. 7d–h).

### RG108 improves insulin resistance via adiponectin expression

**Figure 6:**
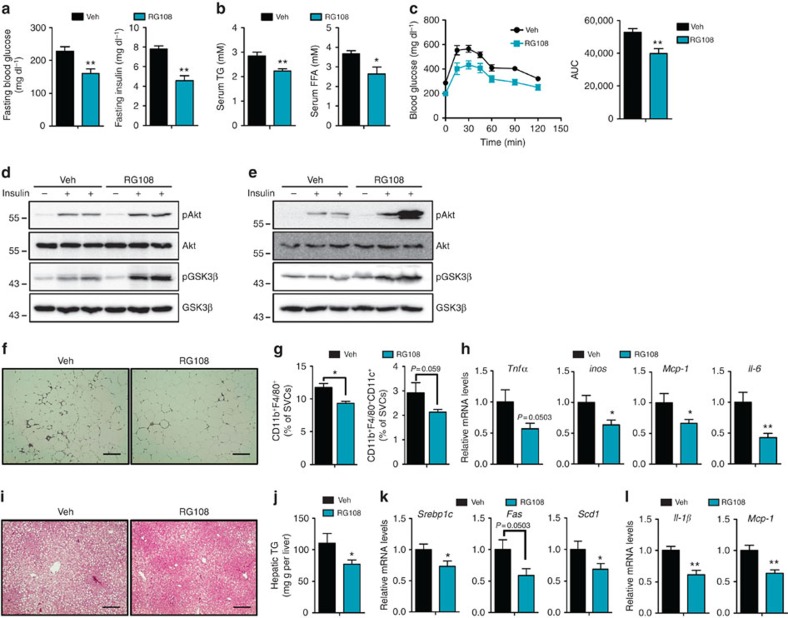
RG108 improves metabolic parameters in *db/db* mice. *db/db* mice were injected with vehicle (Veh, 1% DMSO, *n*=5–7) or RG108 (*n*=5–7). (**a**) Fasting glucose and insulin levels in serum. (**b**) Serum TG and FFA levels. (**c**) OGTT. After 16 h fasting, Veh- or RG108-injected *db/db* and DKO mice were administered 1 g kg^−1^ body weight of glucose bolus by oral gavage and blood glucose levels were monitored. Time course of blood clearance and area under the curve (AUC) are presented. (**d**,**e**) Western blot of insulin signalling in liver (**d**) and skeletal muscle (**e**). Veh- or RG108-injected *db/db* mice were fasted for 16 h, and then 0.75 mU g−^1^ body weight of insulin were intraperitoneally injected. After 30 min, livers and skeletal muscle tissues were collected. (**f**) Histological analysis of epididymal white adipose tissue (eWAT; haematoxylin and eosin (H&E) staining). Scale bar, 200 μm. (**g**) Flow cytometry analysis of SVCs from eWAT. (**h**) mRNA levels of pro-inflammatory genes in SVCs. mRNA levels were measured by qPCR. (**i**) Histological analysis of the liver (H&E staining). Scale bar, 200 μm. (**j**) Hepatic TG contents. (**k**) mRNA levels of lipogenic genes in the liver. mRNA levels were measured by qPCR. (**l**) Relative mRNA levels of inflammatory genes in liver. Results are expressed as the mean±s.e.m. *P<0.05; **P<0.01 in a two-tailed Student's *t-*tests. See Supplementary Fig. 11 for full-length images of blots.

**Figure 7:**
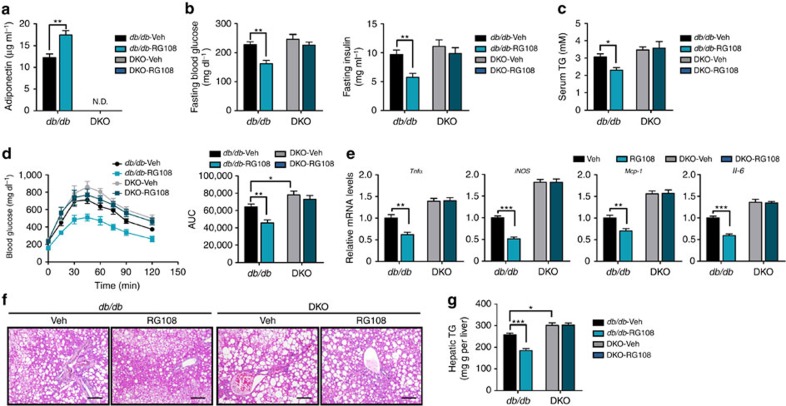
The effects of RG108 on the metabolic parameters abrogated in adiponectin and leptin receptor DKO mice. *db/db* or adiponectin and leptin receptor DKO mice were injected with vehicle (1% DMSO; Veh, *n*=4–5) or RG108 (*n*=4–5). (**a**) Serum adiponectin levels were determined by ELISA. (**b**) Serum fasting glucose and insulin levels. (**c**) Serum TG levels. (**d**) OGTT. After 16-h fasting, mice were intraperitoneally injected with 1 g kg^−1^ body weight of glucose and the blood glucose levels were monitored. Time course of blood clearance and AUC are presented. (**e**) mRNA levels of inflammatory genes were examined in eWAT. mRNA levels were measured by qPCR. (**f**) Histological analysis of the liver (H&E staining). Scale bar, 50 μm. (**g**) Hepatic TG contents. Results are expressed as the mean±s.e.m. *P<0.05; **P<0.01; ***P<0.001 in a two-tailed Student's *t*-tests.

As adiponectin actively regulates systemic energy homeostasis^9^,^15^,^25^, increased levels of adiponectin by RG108 led us to investigate the effects of RG108 on obesity-related metabolic complications. Remarkably, RG108 decreased the levels of fasting glucose, fasting insulin, serum triglycerides (TG) and free fatty acids (FFAs) in *db/db* mice (Fig. 6a,b). RG108 also ameliorated systemic glucose intolerance (Fig. 6c) and insulin signalling cascades in liver and muscle that are major target organs of adiponectin (Fig. 6d,e). Moreover, RG108 mitigated obesity-induced chronic inflammation in adipose tissues (Fig. 6f–h), whereas the DNA methylation degrees at several promoters of inflammation-related genes such as *Pparγ2*, *Tnf*α and *Mcp-1* were not altered by RG108 (Supplementary Fig. 7i–n). Previous studies indicate that adiponectin has anti-inflammatory functions in macrophages^26^,^27^, suggesting that the suppressive effect of RG108 on adipose tissue inflammation would be mediated through adiponectin rather than directly altering the extent of DNA methylation of inflammation-related genes. Furthermore, RG108 alleviated lipid accumulation and expression of lipogenic and pro-inflammatory genes in the liver (Fig. 6i–l). However, RG108 treatment did not lead to significant changes in metabolic parameters in WT lean mice (Supplementary Fig. 8), which might be attributed to the fact that the adiponectin promoter is relatively hypomethylated in lean mouse adipocytes. We also administered RG108 to adiponectin and leptin receptor double knockout (DKO) mice characterized with severely impaired systemic energy homeostasis^28^. Similar to *db/db* mice, RG108 did not affect the body weight and liver functions in DKO mice (Supplementary Fig. 9a–c). In contrast, RG108 failed to induce an increment of serum adiponectin level in DKO mice (Fig. 7a) and the beneficial effects of RG108 on metabolic parameters were abolished in DKO mice (Fig. 7b–g and Supplementary Fig. 9d). These results further demonstrate that RG108 improves obesity-associated metabolic disturbances through the upregulation of adiponectin.

### The R2-binding molecules are altered by inflammation

Because the R2 is newly discovered as one of the regulatory elements of adiponectin, we set out to investigate the potential regulators mediating inflammation-induced suppression of adiponectin expression by isolating the R2-binding proteins from adipocytes incubated with or without TNFα (Supplementary Fig. 10a). Particularly, the binding of certain types of transcription-regulating factors and histone-modifying enzymes were dynamically changed on TNFα challenge (Supplementary Fig. 10b and Supplementary Table 1). These data suggest that obesity-induced repression of adiponectin expression would be attributed to the DNA methylation-mediated suppression of activity of particular element in the adiponectin promoter through controlling the binding of transcriptional regulatory proteins including members of chromatin remodelling complexes.

## Discussion

**Figure 8:**
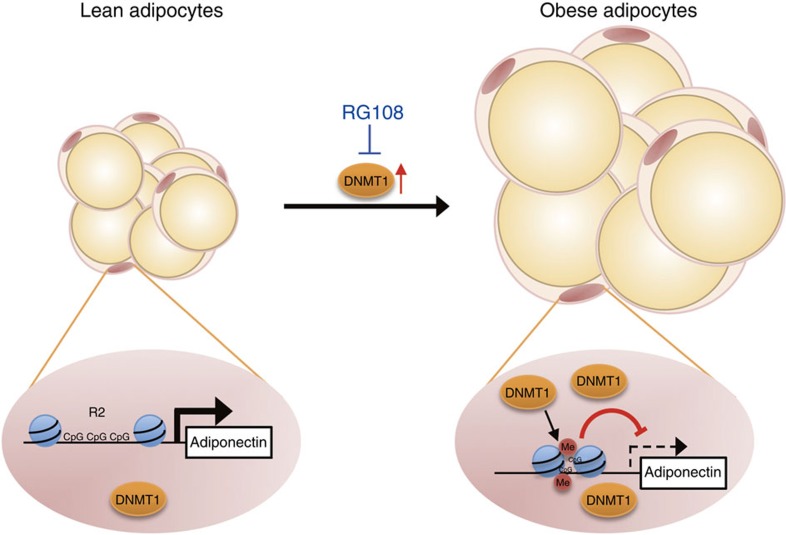
Overall model. In obesity, increased DNMT1 induces DNA hypermethylation at the particular region (R2) of adiponectin promoter, resulting in suppression of adiponectin gene expression in adipocytes.

Despite of the prominent roles of adiponectin in the control of energy balance, the molecular mechanisms involved in obesity-induced dysregulation of adiponectin gene expression have been elusive. The results presented here provide compelling evidence that DNA methylation plays a crucial role in the regulation of adiponectin gene expression, modulating whole-body energy balance in obesity. In adipocytes, DNA methylation at the particular region of adiponectin promoter, the R2, is mediated by DNMT1, and induces the subsequent formation of heterochromatin structure to suppress adiponectin gene expression in obesity. Further, the inhibition of DNMT activity counteracts the downregulation of adiponectin followed by improved metabolic parameters (Fig. 8).

Recent findings have revised the concept of DNA methylation being a stable status except during embryogenesis and carcinogenesis. Instead, DNA methylation appears to be dynamically regulated responding to the changes in nutrient cues, affecting systemic energy homeostasis^29^. Given the previous observations that DNA methylation is one of the key players governing gene expression independently of modulating expression of transcription factors^21^,^30^, we explored the changes of DNA methylation at the adiponectin promoter in obesity where the expression of major transcription factors is retained. Adipocytes isolated from both obese human and mice showed an increase in DNA methylation at the specific CpG motifs, the R2, of adiponectin promoter, concomitantly with the enhanced expression of DNMT1. In obesity, the importance of DNA methylation to suppress adiponectin gene expression has been assessed by using animal models and pharmacological approaches. In *db/db* mice, hypoadiponectinemia was ameliorated by the inhibition of DNMT1 activity by RG108 through reducing DNA hypermethylation at the R2, consequently alleviating metabolic dysregulation. Notably, such beneficial effects were not observed in adiponectin and leptin receptor DKO mice, indicating that adiponectin gene expression is essential for metabolically beneficial effects of RG108 in obese mice. Collectively, these findings provide a novel clue to understand the cause of obesity-induced dysregulation of adiponectin.

Because adipose tissue simultaneously faces interrelated metabolic complications including chronic inflammation, endoplasmic reticulum stress, mitochondrial dysfunction and hypoxia in obesity^31^,^32^, we investigated which factors play critical roles in DNMT1-mediated suppression of adiponectin gene expression in adipocytes, Here we found that pro-inflammatory signalling such as TNFα and IL-1β stimulated DNMT1 expression/activity and enhanced DNA methylation at the R2 in adipocytes, suppressing adiponectin expression. On the other hand, acute treatment of tunicamycin, rotenone or hypoxia potently repressed adiponectin gene expression regardless of DNMT1 expression and subsequent DNA methylation. Nonetheless, these pathophysiological conditions share many players and points of crosstalk ultimately leading to the dysregulation of adipose tissues^31^. Thus, obesity-induced hypermethylation of the R2 *in vivo* is more likely due to the accumulation of pro-inflammatory responses by multi factors involved in the dysregulation of adipose tissues.

In the present study, we found several candidates associated either with the R2 or R2-bound proteins in response to pro-inflammatory stimulation. There were increases in the binding of certain types of transcriptional regulatory proteins engaged in histone modification to the R2. For example, splicing factor proline- and glutamine-rich (SFPQ) and delta 3-like E3 ubiquitin ligase (DTX3L), which were recruited to the R2 on TNFα, are known to be involved in transcriptional repression and chromatin remodelling^33^,^34^. In addition, as suggested by other reports^35^,^36^,^37^, DNMT1 has the ability to methylate selective DNA sequences through forming complexes with specific transcription factors. Thus, it is likely that DNMT1 is guided to the R2 by interacting with particular transcription factor(s) whose binding to the R2 would be altered in obesity. Accordingly, it is clearly of importance to clarify the roles of these newly identified proteins in the obesity-mediated suppression of adiponectin in future studies.

Previous studies have addressed the correlation between human adiponectin single-nucleotide polymorphisms (SNPs) and serum adiponectin levels^38^,^39^,^40^,^41^,^42^. Interestingly, two SNPs (rs17300539 and rs266729), which show significant correlation with serum adiponectin levels^43^,^44^,^45^,^46^, are located within the R2 of the human adiponectin promoter and can serve as CpG dinucleotides as the C is located in the front position of each SNP (Supplementary Fig. 2). For instance, human subjects with a G to A substitution (rs17300539 A/A genotype) exhibit higher plasma adiponectin levels than subjects with the rs17300539 G/G genotype. Likewise, human subjects with a C to G substitution (rs266729 G/G genotype) present a lower adiponectin plasma concentration as compared with subjects with the C/C genotype. Therefore, our findings and the human SNP data strongly support the critical role of DNA hypermethylation in the suppression of adiponectin expression.

Taken together, this study illustrates one of the key regulatory regions in the adiponectin gene whose DNA modification and chromatin remodelling are important for obesity-induced suppression of adiponectin expression. Particularly, these data demonstrate the feasibility of stimulation of adiponectin expression by DNMT inhibitor for the potential therapeutics against obesity-related disease.

## Methods

### Animal experiments

Six or 8-week-old C57BLKS/J-*Lepr*^*db*^*/Lepr*^*db*^ (*db/db*) male, C57BLKS/J-m^+^ male and 8-week-old C57BL/6J male mice were purchased from Central Laboratory Animal Inc. Eight to 10-week-old *db/db* mice and leptin receptor^−/−^/adiponectin^−/−^ DKO male mice with a C57BL/6J background were also used^28^. For the HFD study, 8-week-old mice were fed a NCD or 60% HFD (Research Diet Inc., D12492) for 20 weeks. For the DNMT inhibitor injection study, 8-week-old WT or *db/db* mice were intraperitoneally injected with vehicle (Veh, 1% DMSO (dimethylsulphoxide) in PBS) or RG108 (Cayman, 13302; 5.69 mg kg^−1^ per day) for 32 days. To measure fasting glucose and fasting insulin levels, WT or *db/db* mice were fasted for 16 h and basal blood samples were drawn. For the oral glucose tolerance test (OGTT), WT or *db/db* mice were fasted for 6 or 16 h, respectively, followed by oral gavage of 2 g kg^−1^ or 1 g kg^−1^ glucose bolus. Blood glucose levels were measured 0, 15, 30, 45, 60, 90 and 120 min after oral gavage. For the DNMT inhibitor injection study using *db/db* and DKO mice, 8–10-week-old mice were intraperitoneally injected with Veh or RG108 (5.69 mg kg^−1^·per day) for the first 2 weeks and then increased to 8.2 mg kg^−1^·per day for the remaining 3 weeks. The GTT was performed by intraperitoneal injection of glucose at 1 g kg^−1^ body weight, followed by measurement of blood glucose 0, 15, 30, 45, 60, 75, 90 and 120 min after intraperitoneal injection. All animal experiments were approved by the Seoul National University Animal Experiment Ethics Committee and the Committee on the Use of Live Animals for Teaching and Research of the University of Hong Kong.

### Serum protein and lipid measurements

Serum insulin levels were measured by an enzyme-linked immunosorbent assay (ELISA) (Shibayagi Co. Ltd., AKRIN-001T). Serum alanine aminotransferase and aspartate aminotransferase activities were measured using an activity assay kit (Biovision, K752-100, K753-100 and Sigma Aldrich, MAK052-1KT, MAK055-1KT). Assay kits were used to measure serum TG (Thermo Fischer Scientific Inc., TR22321 and Sigma Aldrich, T2449-10ML) and FFA (Roche, 11 383 174 001). Adiponectin serum levels were measured using a homemade ELISA kit (The University of Hong Kong). Analysis and quantification of adiponectin oligomerization were performed using gel filtration as described previously^47^. In brief, 10 μl of serum was diluted with 1 ml of PBS. Diluted serums were loaded onto an AKTA explorer fast protein chromatography system, fractionated through a Hiload 16/60 Superdex 200 column (GE Healthcare, 28-9893-35) and eluted with PBS at a flow rate of 1 ml min^−1^. Each 0.5-ml fraction was collected and subjected to ELISA analysis.

### Human samples

Human adipose tissue samples were obtained from 12 subjects from the Obesity Research Center at the College of Medicine of King Saud University, Riyadh, Saudi Arabia. The protocol was approved by the College of Medicine Ethics Committee, King Saud University, and written informed consent was obtained from all participants to their enrollment in the study. After adipocyte fractionation and genomic DNA extraction, methylation levels of the adiponectin promoter R2 region were analysed by bisulfite sequencing. Following RNA extraction, RNA was reverse transcribed to complementary DNA (cDNA) and then used in quantitative real-time PCR (qPCR).

### Adipose tissue fractionation and flow cytometry

Epididymal adipose tissues were isolated from each mouse, rinsed in PBS, minced and digested for 35 min at 37 °C in Krebs–Ringer phosphate buffer (pH 7.4) with 2% bovine serum albumin and 1.75 mg ml^−1^ of type I collagenase (Gibco, 17100-017). The digested tissue was filtered through a 250-μm nylon mesh to remove undigested tissue and centrifuged at 3,000 r.p.m. for 5 min. The floating adipocyte fraction and SVC pellet were washed several times. Flow cytometry analysis of adipose tissue macrophage was performed as described previously^48^. In brief, SVC pellets were incubated with red blood cell lysis buffer (eBioscience, 00-4333-57) for 5 min. After washing twice with PBS, SVC pellets were incubated with CD16/32 (eBioscience, 14-0161), as blocking antibody, for 10 min at 4 °C to staining with fluorescence-labelled primary antibodies for 30 min at 4 °C. Cells were gently washed with and resuspended in PBS. SVCs were analysed using the fluorescence-activated cell sorting CantoII instrument (BD Bioscience). The following antibodies were used for flow cytometry analysis—anti-CD11b (eBioscience, 27-0112-81, 1:50 dilution), anti-F4/80 (eBioscience, 45-4801-80, 1:50 dilution) and anti-CD11c (eBioscience, 12-0114-82, 1:50 dilution).

### Genomic DNA purification and bisulfite sequencing

DNA was purified by phenol–chloroform extraction. Cells were lysed for 1 h in lysis buffer (50 mM Tris-Cl (pH 8.0), 50 mM ethylenediaminetetraacetic acid (EDTA; pH 8.0), 100 mM NaCl, 2% SDS and 20 μg ml^−1^ RNase) at 37 °C and digested with 10 μg ml^−1^ proteinase K for 3 h at 50 °C. Bisulfite conversion was performed using the EpiTect Bisulfite Kit (Qiagen, 59104). Converted DNA was amplified by PCR using primers designed with Methprimer software (www.urogene.org/methprimer/index1.html). PCR conditions were 95 °C for 7 min and 35 cycles of 95 °C 1 min, 55 °C 30 s and 72 °C 1 min, followed by 10 min at 72 °C. PCR products cloned into bacteria using the TOPO TA cloning kit (Invitrogen, 450641). Eight clones for each sample were sequenced using COSMO Genetech commercial services. The primer sequences that were used for PCR are provided in Supplementary Table 2.

### Cell culture and transient transfection

3T3-L1 cells were obtained from ATCC (CL-173). 3T3-L1 cells were grown to confluence in Dulbecco's modified Eagle medium (DMEM; Hyclone, SH30243.01) supplemented with 10% bovine calf serum (Gibco, 26010-074). To induce adipocyte differentiation, at 2 days post confluence, 3T3-L1 cells were incubated with DMEM containing 10% fetal bovine serum (FBS; Hyclone, SH30919.03), 0.52 mM 3-isobutyl-1-methylxanthine (Sigma Aldrich, I5879), 1 μM dexamethasone (Sigma Aldrich, D1756) and 1 μg ml^−1^ insulin (Roche, 11 376 497 001) for 2 days. Then, the culture medium was replaced with DMEM containing 10% FBS and 1 μg ml^−1^ insulin and the cells were cultured for 2 additional days. The culture medium was changed every 2 days with DMEM containing 10% FBS.

Small interfering RNA duplexes were designed and purchased from Bioneer (DNMT1, 1350629; DNMT3a, 1350650). pcDNA3-DNMT1 vector was kindly donated by Dr Francois Fuks (Free University of Brussels, Belgium). Each small interfering RNA (20 μM), pcDNA3.1-myc-His and pcDNA3.1-DNMT1 were delivered into 3T3-L1 cells using Microporator (Digital Bio Technology Co. Ltd.).

For the various pathological environmental-mimicking experiments, differentiated 3T3-L1 adipocytes were incubated with or without 10 ng ml^−1^ TNFα (R&D Systems, 210-TA), 10 ng ml^−1^ IL-1β (R&D Systems, 201-LB), 1 μg ml^−1^ tunicamycin (Calbiochem, 654080), 1 μM rotenone (Sigma Aldrich, R8875) or incubated in normal or hypoxic (1% O_2_ concentration) conditions for 24 h. To inhibit DNMT1 activity, 3T3-L1 adipocytes were preincubated with RG108 (100 μM) for 24 h before treatment with TNFα.

### RNA isolation and qPCR

The RNA isolation and cDNA synthesis procedure were performed as described previously^49^. In brief, total RNA was isolated from mouse cells or cell lines with TRIzol Reagent (Ambion, 15596-018) and subjected to cDNA synthesis using Maxima First Strand cDNA synthesis Kit for qPCR (Thermo Scientific, K1642). RNA from purified human adipocytes was extracted using the RNeasy Lipid Tissue kit (Qiagen, 74804), and subjected to cDNA synthesis using the High Capacity RNA-to-cDNA kit (Applied Biosystems, 4387406). mRNA relative amounts were measured using the CFX96 Real-Time System (Bio-Rad Laboratories Inc.) and calculated by normalization to the level of cyclophilin mRNA (mouse cells or cell lines), or to the level of *GAPDH* mRNA (human cells). The primer sequences that were used for quantitative real-time PCR analyses are provided in Supplementary Table 3.

### Restriction enzyme accessibility assay

The restriction enzyme accessibility assay has been described previously^50^. In brief, adipocytes from epididymal adipose tissue and 3T3-L1 cells were harvested with RSB buffer (10 mM Tris-HCl (pH 7.4), 10 mM NaCl, 5 mM MgCl_2_, 0.1% Nonidet P-40 (NP-40), 5 mM butyrate, 10 mM NaF and 1 mM NaVO_7_, supplemented with protease inhibitors) and incubated for 20 min on ice. The cell pellets were homogenized through 27-gauge syringes, centrifuged at 2,000 r.p.m. at 4 °C for 5 min and resuspended in 100 μl of fresh RSB buffer. Resuspended gDNA was digested with 100 U of AluI, EcoRI and BamHI (TaKaRa, 1004A, 1040A and 1010A). Reactions were stopped by adding proteinase K and 2% SDS for 6 h at 45 °C. Chromosomal DNA was then extracted with phenol–chloroform twice, precipitated with isopropanol and resuspended in distilled water. The precipitated DNA was amplified by PCR. Relative amounts of PCR product were measured using the CFX96 Real-Time System (Bio-Rad Laboratories Inc.) The primer sequences that were used for PCR are provided in Supplementary Table 2.

### Chromatin immunoprecipitation

3T3-L1 cells were crosslinked in 1% formaldehyde at room temperature for 10 min, and crosslinking was terminated by the addition of glycine (125 mM) for 2 min. After a rinse with PBS, cells were collected with buffer 1 (100 mM Tris-HCl (pH 9.4) and 10 mM dithiothreitol (DTT)). For the isolation of crude nuclei, cell pellets were resuspended with buffer 2 (10 mM Tris-HCl (pH 8.0), 0.25% Triton-X-100, 0.5% NP-40, 10 mM EDTA, 0.5 mM EGTA and 1 mM DTT) for 10 min and precipitated with centrifugation. Collected nuclei were washed with buffer 3 (10 mM Tris-HCl (pH 8.0), 0.2 M NaCl, 1 mM EDTA, 0.5 mM EGTA and 1 mM DTT) twice and resuspended in buffer 3 without NaCl for sonication. After sonication, equal amounts of chromatin solution were incubated in 1 × radioimmunoprecipitation assay buffer with antibodies at 4 °C overnight. The immunoprecipitates were collected by adding protein A-sepharose beads and sequentially washed with modified TSE (mTSE) 1 (0.1% SDS, 0.5% Triton-X-100, 2 mM EDTA, 20 mM Tris-HCl (pH 8.0), and 100 mM NaCl), mTSE2 (0.1% SDS, 0.5% Triton-X-100, 2 mM EDTA, 20 mM Tris-HCl (pH 8.0) and 200 mM NaCl), mTSE3 (0.25 M LiCl, 0.5% NP-40, 1 mM EDTA, 10 mM Tris-HCl (pH 8.0)) and TE (10 mM Tris-HCl (pH 8.0) and 1 mM EDTA) buffers. Then, immune complexes were eluted with elution buffer (1% SDS and 0.1 M NaHCO_3_), and the protein–DNA crosslinking was reversed by incubating at 65 °C for 12 h with 200 mM of NaCl. DNA was extracted with phenol/chloroform and precipitated with ethanol and 20 μg of glycogen. Relative amounts of PCR product were measured using the CFX96 Real-Time System (Bio-Rad Laboratories Inc.) The primer sequences that were used for PCR are provided in Supplementary Table 2. The following antibodies were used for chromatin immunoprecipitation—anti-DNMT1 (ab92453, used 5 μg for 20 μg chromatin), anti-MeCP2 (ab2828, used 5 μg for 20 μg chromatin) and anti-H2K9Ac (ab4441, used 1 μg for 20 μg chromatin) from Abcam.

### Western blotting

Cells and tissues were lysed in TGN buffer (150 mM NaCl, 50 mM Tris-HCl (pH 7.5), 0.2% NP-40, 1 mM phenylmethylsulfonyl fluoride, 100 mM NaF, 1 mM Na_3_VO_4_, 10 μg ml^−1^ aprotinin, 2 μg ml^−1^ pepstatin A and 10 μg ml^−1^ leupeptin) and radioimmunoprecipitation assay buffer, respectively. Proteins were separated by electrophoresis on SDS–polyacrylamide gels and transferred to polyvinylidene difluoride membranes (Millipore Corp., IPVH00010). After transfer, the membranes were blocked with 5% non-fat milk and probed with primary antibodies. Antibodies against adiponectin (Cell Signaling, 2789, 1:1,000 dilution), β-actin (Sigma Aldrich, A5316, 1: 2,000 dilution), PPARγ (Santa Cruz Biotechnology Inc., 7,196, 1:1,000 dilution), DNMT1 (Abcam, ab13537, 1:500 dilution), AKT (Cell Signaling, 9,272, 1:1,000 dilution), pAKT (Cell Signaling, 4051, 1:1,000 dilution), GSK3β (BD Sciences, 610201, 1:1,000 dilution) and pGSK3β (Cell Signaling, 9,336, 1:1,000 dilution) were used. The bands were visualized with horseradish peroxidase-conjugated secondary anti-rabbit IgG or anti-mouse IgG antibodies (Sigma Aldrich, A0545 and A9044, respectively) and enhanced chemiluminescence. When liver and muscle tissues were collected from insulin-treated mice, the mice were fasted for 16 h, and then 0.75 mUg^−1^ body weight of insulin was intraperitoneal injected. Liver and muscle tissues were collected 30 min later.

### DNMT enzymatic activity assay

To purify nuclear extracts, cells were collected in hypotonic buffer (50 mM KCl, 25 mM HEPES (pH 7.8), protease inhibitor cocktail (GeneDEPOT, P3100)) with 0.5% NP-40. Collected cells were incubated on ice for 10 min and washed with hypotonic buffer without NP-40 twice. After removing the supernatant, pellets were resuspended with nucleus extraction buffer (500 mM KCl, 10% glycerol, 25 mM HEPES (pH 7.8), protease inhibitor cocktail) and incubated for 5 min. The supernatant was collected for nuclear protein assessment after centrifugation. The DNMT enzymatic activity assay was performed using the EpiQuik DNA Methyltransferase Activity/Inhibition Assay Kit (Epigentek Group Inc., P-3001) with nuclear extract.

### Purification and characterization of the R2-binding protein

To obtain the nuclear extracts, vehicle- or TNFα-treated differentiated 3T3-L1 cells were washed twice with cold PBS and treated with hypertonic buffer A (50 mM KCl, 0.5% NP-40, 25 mM HEPES (pH 7.8) and protease inhibitor cocktail) for 5 min on ice. After centrifugation, the nuclei were washed twice with hypotonic buffer A without NP-40. The pellets were resuspended with high salt buffer B (500 mM KCl, 25 mM HEPES (pH 7.8), 10% glycerol and protease inhibitor cocktail) and pipetted several times. After centrifugation, the supernatant was collected and used for pull-down assay. We designed the oligonucleotides containing the R2. After synthesis, the oligonucleotides were biotinylated and annealed according to the manufacturer's protocol (Thermo Scientific, 89818). The oligonucleotide duplex was incubated with nuclear extracts for overnight at 4 °C. Complex of the oligonucleotides and protein was precipitated by streptavidin-coated bead (Thermo Scientific, 20359). The oligonucleotide-binding proteins were obtained using on-bead digestion methods^51^. These were then subjected to mass spectrometry. The following oligonucleotide sequences were used for pull-down assay; forward (f), 5′-GATTCACGATTTAATTCAAAAGCTTTGTGCTCCCGAGAATCAGCTCTGGTCTTTCAAAAATAAGATGTGAGTCCGCCGAGAGGCTCCCAAGGTATTGCCTTGCCAAC-3′; reverse (r), 5′-GTTGGCAAGGCAATACCTTGGGAGCCTCTCGGCGGACTCACATCTTATTTTTGAAAGACCAGAGCTGATTCTCGGGAGCACAAAGCTTTTGAATTAAATCGTGAATC-3′.

### Statistical analysis

All the results are presented as mean±s.e.m. Statistical significance was assessed by the two-tailed Student's *t*-test using GraphPad Prism 5.0 (GraphPad Software). There was no difference in variances for all comparisons in F-test. When cells were used for experiments, three replicates per group were chosen. All *n* values defined in the legends refer to biological replicates unless otherwise indicated. In mouse experiments requiring technical manipulation, at least five mice were used per group. If technical failures such as failure of oral gavage and intraperitoneal injection occurred, those samples were excluded from the final analysis. Differences were considered statistically significant at *P*<0.05. No statistical method was used to predetermine sample size. The experiments were not randomized. The investigators were not blinded to allocation during experiments and outcome assessment.

## Additional information

**How to cite this article:** Kim, A.Y. *et al.* Obesity-induced DNA hypermethylation of the adiponectin gene mediates insulin resistance. *Nat. Commun.* 6:7585 doi: 10.1038/ncomms8585 (2015).

## Supplementary Material

Supplementary Information Supplementary Figures 1-11 and Supplementary Tables 1-3
