# Supplementary material for: Obesity-induced DNA hypermethylation of the adiponectin gene mediates insulin resistance
Source: Nat Commun. 2015 Jul 3;6:7585. doi: 10.1038/ncomms8585 (PMC4506505; doi:10.1038/ncomms8585)
Supplement: Supplementary Information — Supplementary Figures 1-11 and Supplementary Tables 1-3 [file ncomms8585-s1.pdf]

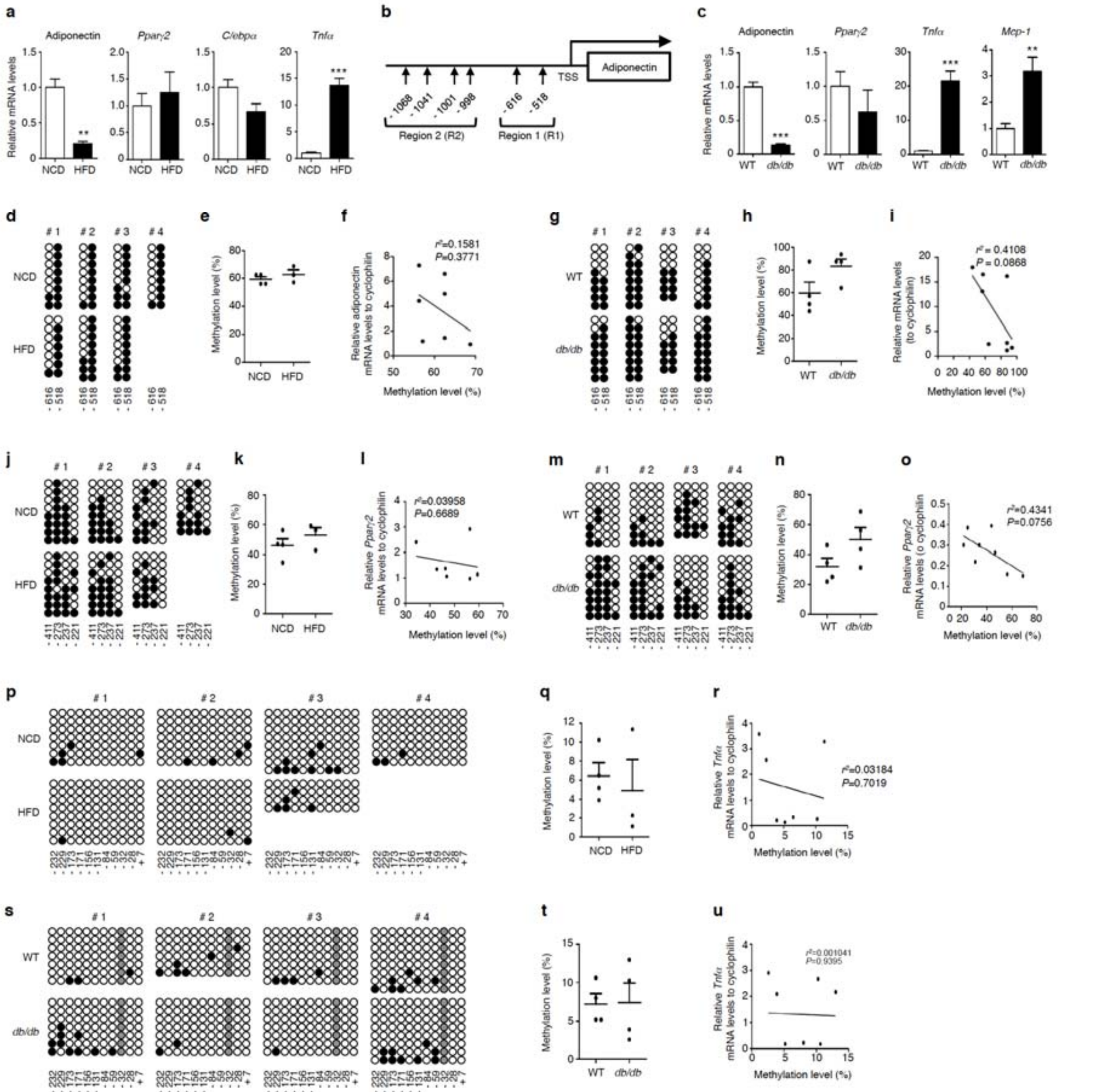

Supplementary Figure 1. DNA methylation of the adiponectin promoter R1, *Pparg2*, and *Tnfa* promoter in adipocytes is not affected by obesity.

(**a**) Relative amounts of adiponectin, *Pparγ2*, *C/ebpα*, and *Tnfα* mRNA in adipocytes of NCD-fed (n = 4) or HFD-fed (n = 3) mice. (**b**) Schematic overview of the CpG dinucleotide regions on the adiponectin promoter. (**c**) Relative mRNA levels of adiponectin, *Pparγ2*, *Tnfα*, and *Mcp-1* in adipocytes from WT (n = 4) or *db/db* mice (n = 4). (**d**) Bisulfite sequencing analysis of the adiponectin promoter R1 in adipocytes from NCD-fed (n = 4) or HFD-fed (n = 3) mice. (**e**) Quantification of the bisulfite sequencing results from (**d**). (**f**) Correlation between the R1 DNA methylation and adiponectin mRNA levels in adipocytes from NCD-fed (n = 4) or HFD-fed (n = 3) mice. (**g)** Bisulfite sequencing analysis of the adiponectin promoter R1 region in adipocytes of WT (n = 4) or *db/db* (n = 4) mice. (**h**) Quantification of the bisulfite sequencing results from (**g**). (**i**) Correlation between the R1 DNA methylation and adiponectin mRNA levels in adipocytes from WT (n = 4) or *db/db* (n = 4) mice. (**j**) Bisulfite sequencing analysis of *Pparγ2* promoter in adipocytes from NCD-fed (n = 4) or HFD-fed (n = 3) mice. (**k**) Quantification of the bisulfite sequencing results from (**j**). (**l**) Correlation between DNA methylation and *Pparγ2* mRNA levels of in adipocytes from NCD-fed (n = 4) or HFD-fed (n = 3) mice. (**m**) Bisulfite sequencing analysis of *Pparγ2* promoter in adipocytes from WT (n = 4) or *db/db* (n = 4) mice. (**n**) Quantification of the bisulfite sequencing results from (**m**). (**o**) Correlation between DNA methylation and *Pparγ2* mRNA levels in adipocytes

from WT (n = 4) or *db/db* (n = 4) mice. (**p**) Bisulfite sequencing analysis of *Tnfα* promoter in adipocytes from NCD-fed (n = 4) or HFD-fed (n = 3) mice. (**q**) Quantification of the bisulfite sequencing results from (**p**). (**r**) Correlation between DNA methylation and *Tnfα* mRNA levels in adipocytes from NCD-fed (n = 4) or HFD-fed (n = 3) mice. (**s**) *Tnfα* promoter bisulfite sequencing analysis in adipocytes from WT (n = 4) or *db/db* (n = 4) mice. Grey circle indicates the C, which is followed by A instead of G. (**t**) Quantification of the bisulfite sequencing results from (**s**). (**u**) Correlation between DNA methylation and *Tnfα* mRNA levels in adipocytes from WT (n = 4) or *db/db* (n = 4) mice. Results are expressed as mean ± SEM. #; individual mice.

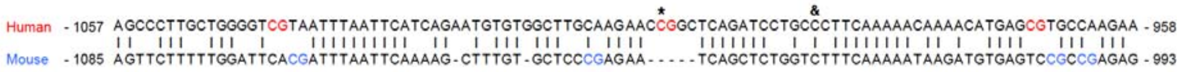

Supplementary Figure 2. Alignment of the human and mouse R2 DNA sequences.
*, site of human adiponectin SNP rs17300539; &, site of human adiponectin SNP rs266729.

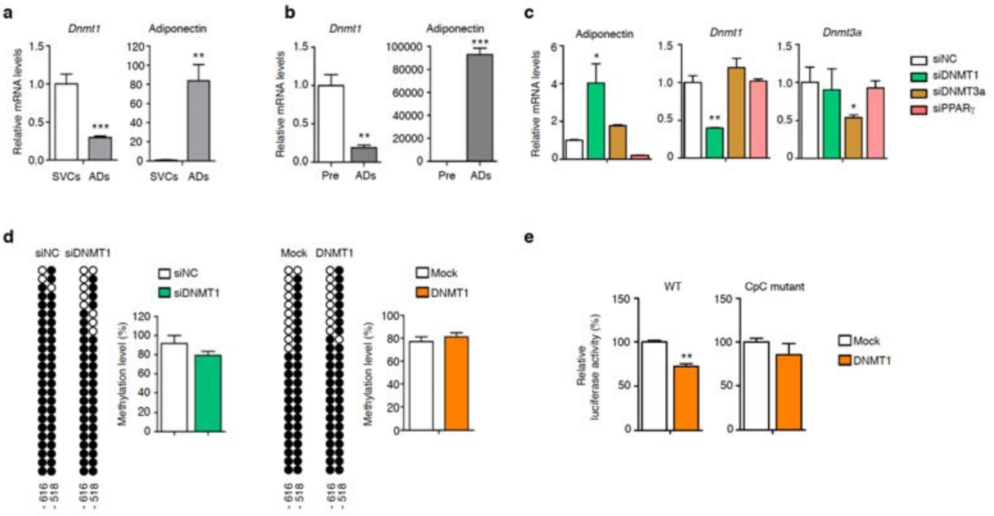

Supplementary Figure 3. DNMT1-mediated R2 DNA methylation suppresses adiponectin gene expression.

(**a**) Relative mRNA levels of *Dnmt1* and adiponectin in SVCs or adipocytes (ADs) obtained from mouse epididymal adipose tissue (n = 5). (**b**) mRNA expression of *Dnmt1* and adiponectin in preadipocytes (Pre) or differentiated 3T3-L1 adipocytes (ADs) (n = 3). (**c**) mRNA levels of adiponectin, *Dnmt1*, and *Dnmt3a* in negative control (NC), DNMT1, DNMT3a, or PPARγ knocked down 3T3-L1 cells (n = 3). (**d**) R1 DNA methylation levels in DNMT1-suppressed or -overexpressed 3T3-L1 cells (n = 3). (**e**) Reporter assay with WT or mutant form of adiponectin promoter, which is all the CpGs are substituted to CpCs at the R2, in 3T3-L1 adipocytes. Results are expressed as mean ± SEM. Similar results were obtained at least more than three independent. **P* < 0.05; ***P* < 0.01; ****P* < 0.001 in a two-tailed Student's *t-test*.

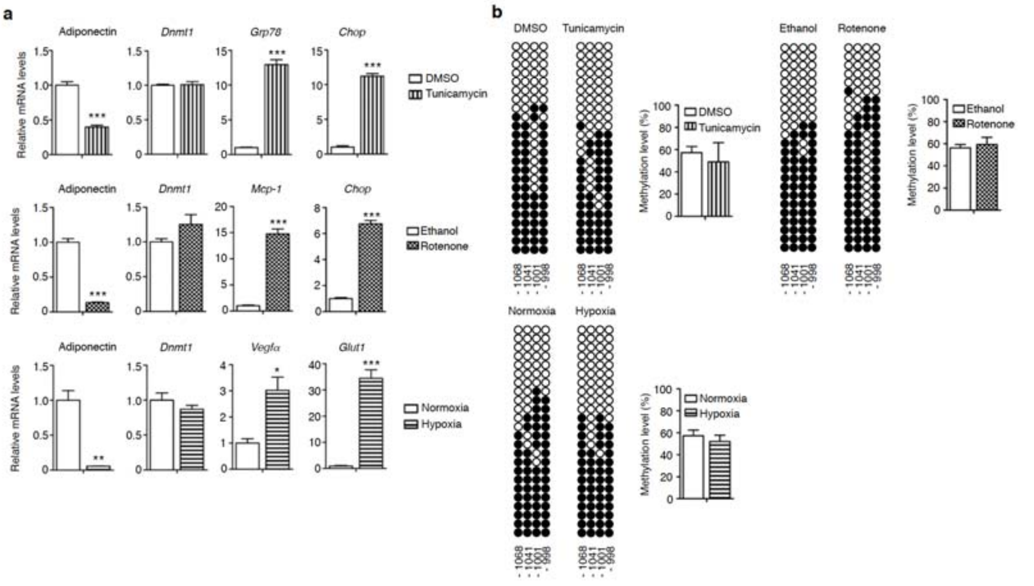

Supplementary Figure 4. Reduction of adiponectin gene expression induced by ER stress, mitochondrial dysfunction, and hypoxia is not accompanied by modification of R2 DNA methylation.

(**a**) Differentiated 3T3-L1 cells were incubated with or without 1 mg/mL tunicamycin, 1 mM rotenone, or incubated in normal or hypoxic (1% O_2 concentration) conditions for 24 h. mRNA levels of adiponectin, *Dnmt1*, *Grp78*, *Chop*, *Mcp-1*, *Vegf*α, and *Glut1* were measured (n = 3). (**b**) DNA methylation levels of the adiponectin promoter R2 in 3T3-L1 cells. Degrees of DNA methylation were quantified (n = 3). Results are expressed as mean ± SEM. Similar results were obtained at least more than three independent. *P < 0.05; **P < 0.01; ***P < 0.001 in a two-tailed Student's *t-test*.

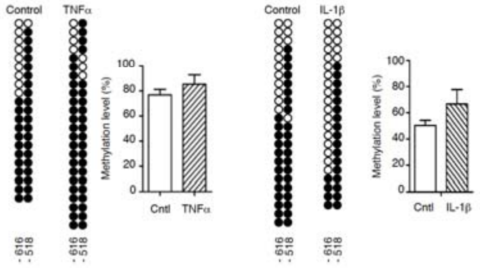

Supplementary Figure 5. Inflammatory cytokines do not influence R1 DNA methylation. Differentiated 3T3-L1 cells were incubated with or without 10 ng/mL TNFα or 10 ng/mL IL-1β for 24 h. Bisulfite sequencing analysis of the adiponectin promoter R1 and quantification of bisulfite sequencing results (n = 3). Results are expressed as mean ± SEM. Similar results were obtained at least more than three independent. Cntl; control.

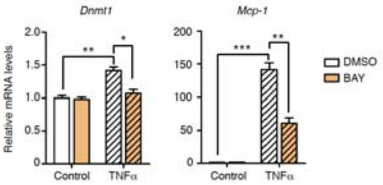

Supplementary Figure 6. TNFα-induced DNMT1 gene expression is blocked by inhibition of NF-κB.

Differentiated 3T3-L1 adipocytes were incubated with or without 10 mM of BAY-11-7082 (BAY) for 6 hr, then were incubated another 24 hr with or without TNFα. Results are expressed as the mean ± SEM. Similar results were obtained at least more than three independent. * $P < 0.05$; ** $P < 0.01$; *** $P < 0.001$ in a two-tailed Student's *t-test*.

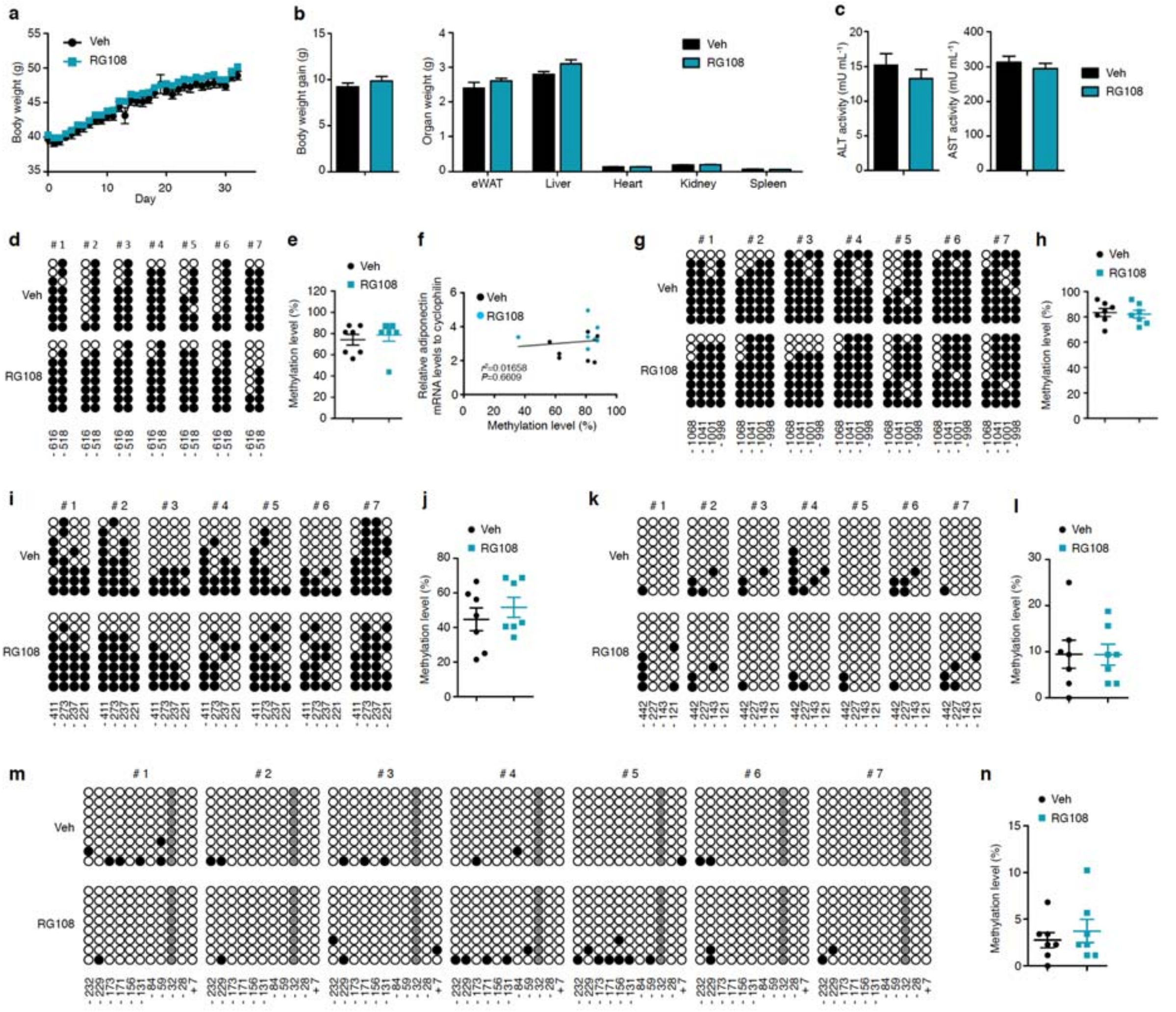

Supplementary Figure 7. RG108 does not influence body weight, organ weight, and DNA methylation at the R1 in adipocytes, at the R2 in the liver or at the promoters of several genes related with inflammation in SVCs of *db/db* mice.

Eight-week-old *db/db* mice were intraperitoneally (i.p.) injected with Veh (n = 5-7) or RG108 (5.68 mg·kg^{-1}·day^{-1}, n = 5-7) for 32 days. (**a**) Body weight changes during the experimental period. (**b**) Body weight gain and organ mass. eWAT, epididymal white adipose tissue. (**c**) Serum ALT and AST activities. (**d**,**e**) DNA methylation analysis of the adiponectin promoter R1 in adipocytes. (**d**) Bisulfite sequencing analysis and quantification of the results. (**f**) Correlation between adiponectin mRNA levels in adipocytes and R1 DNA methylation levels. r^2 and *P*-values are indicated on the graph. (**g**,**h**) DNA methylation analysis of the adiponectin promoter R2 in the liver. (**i**) Bisulfite sequencing results of *Ppar*γ*2* promoter in SVCs of Veh (n = 7) or RG108 (n = 7) injected *db/db* mice. (**j**) Percentage of DNA methylation at the *Ppar*γ*2* promoter. (**k**) DNA methylation data of *Mcp-1* promoter in SVCs of Veh (n = 7) or RG108 (n = 7) injected *db/db* mice. (**l**) Percentage of DNA methylation at the *Mcp-1* promoter. (**m**) DNA methylation data of *Tnf*α promoter in SVCs of Veh (n = 7) or RG108 (n = 7) injected *db/db* mice. Grey circle indicates the C, which is followed by A instead of G. (**n**) Percentage of DNA methylation at the *Tnf*α promoter. Results are expressed as mean ± SEM. #; individual mice.

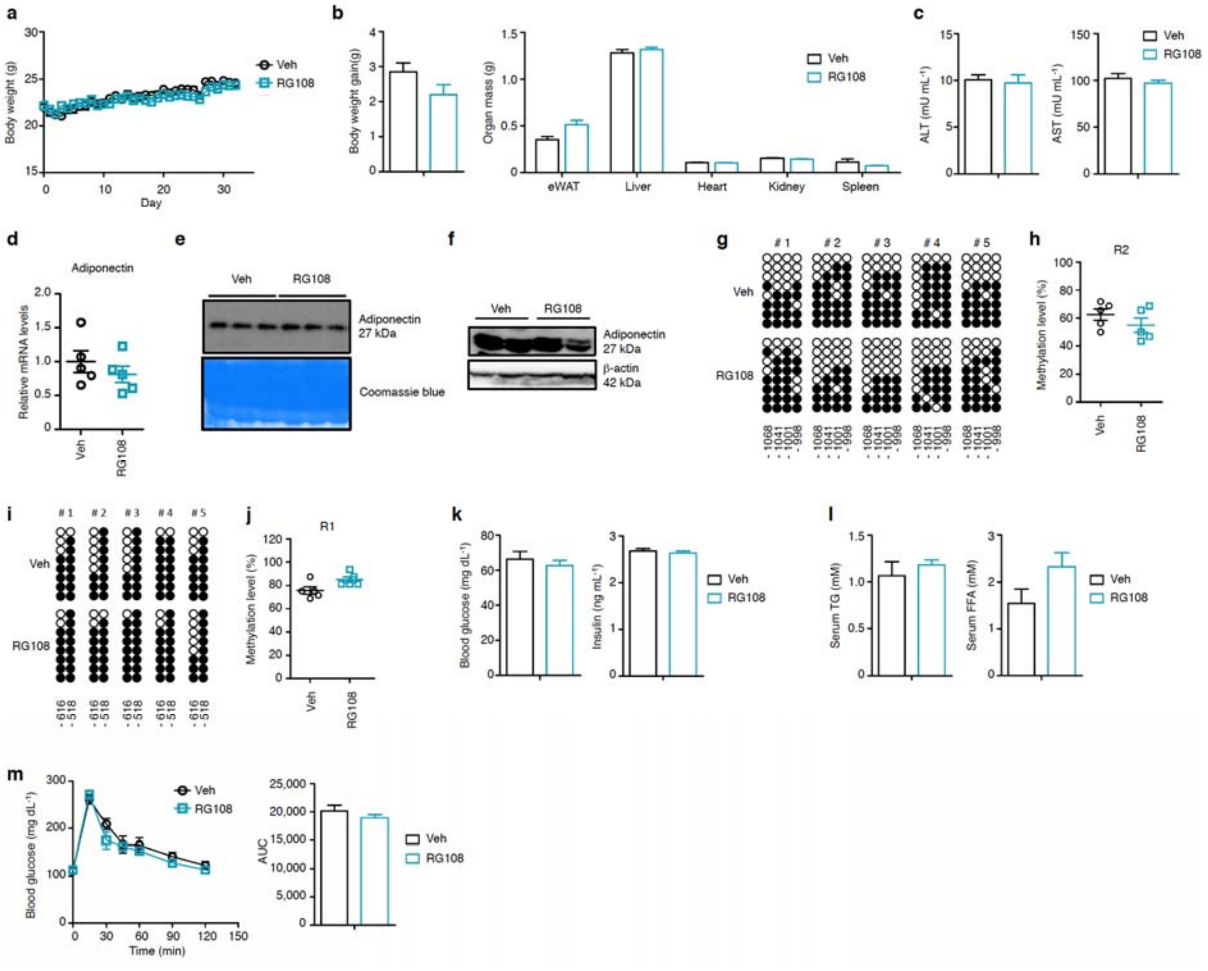

Supplementary Figure 8. RG108 does not influence any metabolic parameter in WT mice.
One percent DMSO (Veh, n = 5) or RG108 (5.69 mg·kg^{-1}·day^{-1}, n = 5) were administered to 8-week-old WT mice for 32 days. (**a**) Body weight change. (**b**) Body weight gain and organ mass. eWAT, epididymal white adipose tissue. (**c**) Serum ALT and AST activities. (**d**), Relative adiponectin mRNA levels in adipocytes. (**e**) Serum adiponectin levels. (**f**) Western blotting of adiponectin in eWAT. (**g**–**j**) Bisulfite sequencing analysis of the adiponectin promoter R2 and R1. (**k**)**,** Fasting blood glucose and fasting serum insulin levels. (**l**) Serum TG and FFA levels. (**m**) Oral glucose tolerance test. After 6 hr fasting, Veh- or RG108-injected WT mice were administrated 2 g/kg body weight of glucose bolus by oral gavage and blood glucose levels were monitored. Time course of blood clearance and area under the curve (AUC) are presented. Results are expressed as mean ± SEM. #; individual mice.

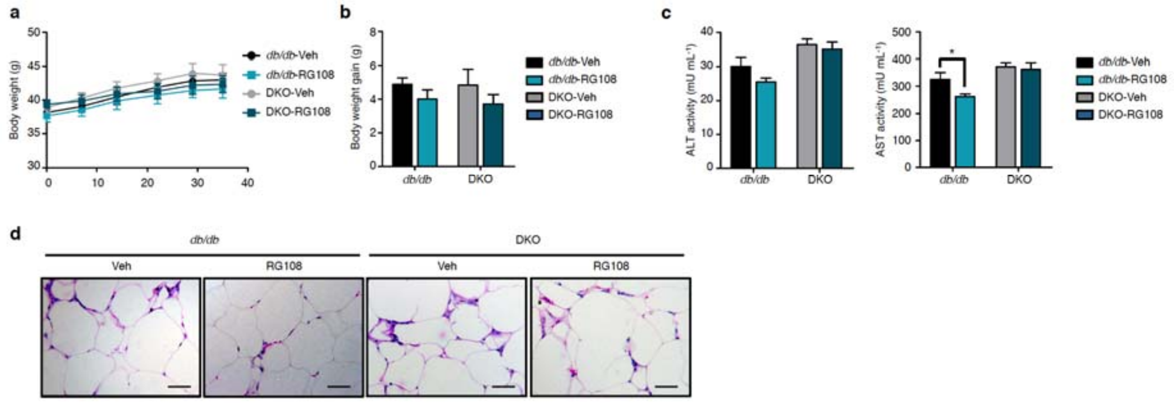

Supplementary Figure 9. RG108 does not influence the body weight, organ weight, and liver function in *db/db* and adiponectin and leptin receptor knockout (DKO) mice and cannot suppress adipose tissue inflammation in DKO mice.

Eight to ten-week-old *db/db* or adiponectin and leptin receptor double knockout (DKO) mice were injected with Veh (n = 4–5) or RG108 (n = 4–5). (**a**) Body weight changes during the experimental period. (**b**) Body weight gain. (**c**) Serum ALT and AST activities. (**d**) H&E stained sections from WAT. Scale bar: 50 mm. Results are expressed as mean ± SEM. *P < 0.05 in a two-tailed Student's *t-test*.

a

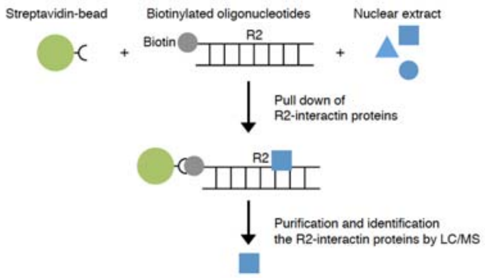

b

Increased binding by TNFα	Decreased binding by TNFα
SFPQ	BAZ1B
DTX3L	FBRL
EF1A1	RBP2
KHDR1	PRP8
RBM25	NOP58
ELAV1	DDX41
HNRH2	DDX52
	UP20

Supplementary Figure 10. Identification of the R2 binding molecules
(**a**) Experimental scheme of oligonucleotide pull down assay. (**b**) List of binding proteins whose affinity was influenced by pro-inflammatory signals. The R2 binding molecules were obtained from three independent experiments.

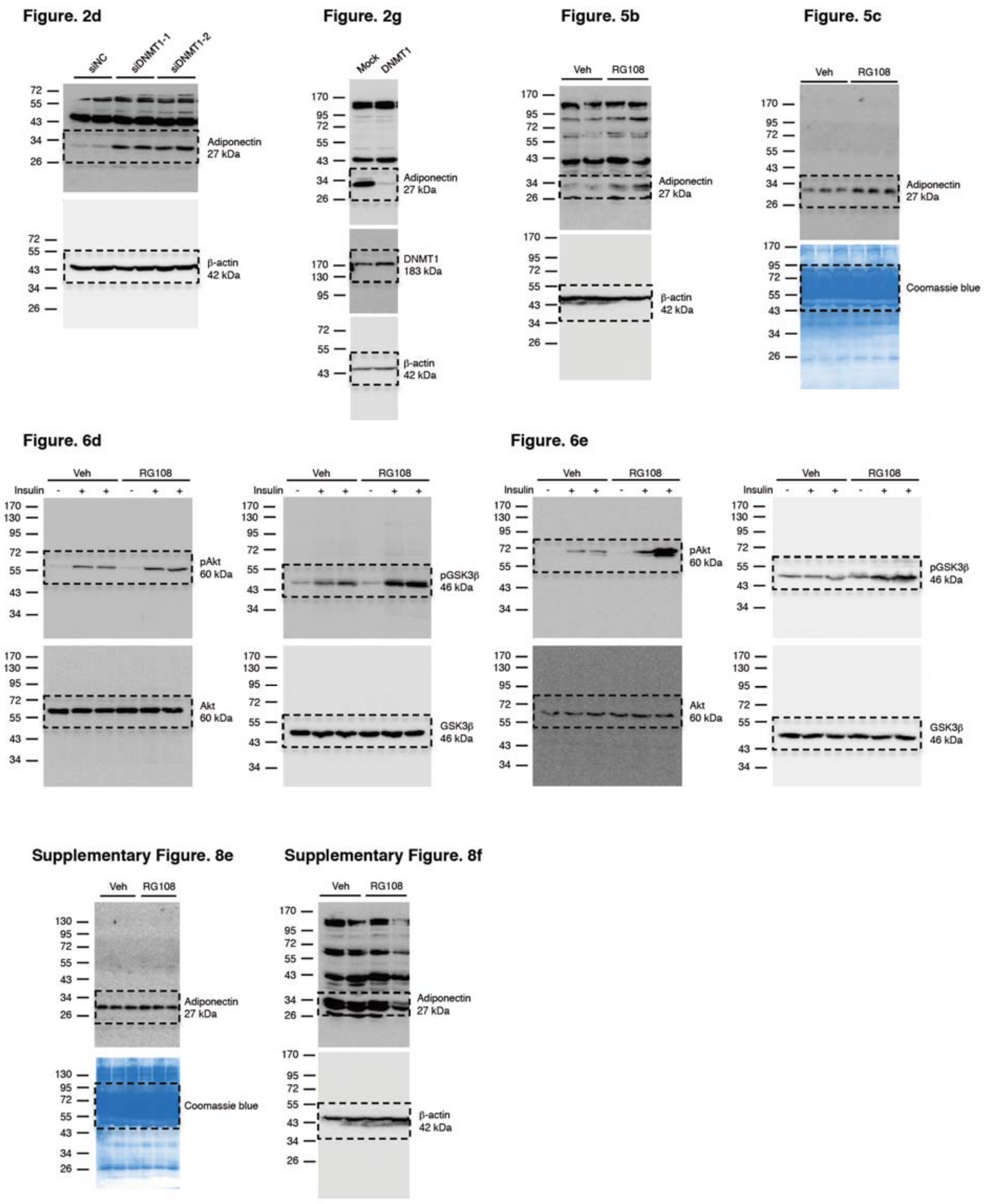

Supplementary Figure 11. Full-length images of blots and membrane presented in main figures and supplementary figures

Proteins that increased binding to the R2 by TNFα

Protein	Functions
SFPQ	DNA- and RNA binding protein, involved in several nuclear processes. SFPQ is involved in transcriptional regulation. Transcriptional repression is probably mediated by an interaction of SFPQ with SIN3A and subsequent recruitment of histone deacetylases (HDACs). The SFPQ-NONO-NR5A1 complex binds to the CYP17 promoter and regulates basal and cAMP-dependent transcriptional activity. SFPQ isoform Long binds to the DNA binding domains (DBD) of nuclear hormone receptors, like RXRA and probably THRA, and acts as transcriptional corepressor in absence of hormone ligands.
DTX3L	Ubiquitin ligase that mediates monoubiquitination of 'Lys-91' of histone H4 (H4K91ub1), in response to DNA damage. The exact role of H4K91ub1 in DNA damage response is still unclear but it may function as a licensing signal for additional histone H4 post- translational modifications such as H4 'Lys-20' methylation (H4K20me). In concert with PARP9, plays a role in PARP1-dependent DNA damage repair. PARP1-dependent PARP9-DTX3L-mediated ubiquitination promotes the rapid and specific recruitment of 53BP1/TP53BP1, UIMC1/RAP80, and BRCA1 to DNA damage sites
EF1A1	Positively regulates the transcription of MTUS1 and negatively regulates the transcription of MTUS2/TIP150. With EEF1A1 and TXK, forms a complex that acts as a T-helper 1 (Th1) cell-specific transcription factor and binds the promoter of IFN- gamma to directly regulate its transcription. Involved in the base excision repair (BER) pathway, by catalyzing the poly ADP-ribosylation of a limited number of acceptor proteins involved in chromatin architecture and in DNA metabolism. Required for PARP9 and DTX3L recruitment to DNA damage sites. PARP1-dependent PARP9-DTX3L-mediated ubiquitination promotes the rapid and specific recruitment of 53BP1/TP53BP1, UIMC1/RAP80, and BRCA1 to DNA damage sites.
KHDR1	Recruited and tyrosine phosphorylated by several receptor systems, for example the T-cell, leptin and insulin receptors.. Represses CBP-dependent transcriptional activation apparently by competing with other nuclear factors for binding to CBP. Also acts as a putative regulator of mRNA stability and/or translation rates and mediates mRNA nuclear export.
RBM25	RNA-binding protein that acts as a regulator of alternative pre-mRNA splicing. Involved in apoptotic cell death through the regulation of the apoptotic factor BCL2L1 isoform expression. When overexpressed, stimulates proapoptotic BCL2L1 isoform S 5'-splice site (5'-ss) selection, whereas its depletion caused the accumulation of antiapoptotic BCL2L1 isoform L.
ELAV1	RNA-binding protein that binds to the 3'-UTR region of mRNAs and regulates their stability.
HNRH2	This protein is a component of the heterogeneous nuclear ribonucleoprotein (hnRNP) complexes which provide the substrate for the processing events that pre-mRNAs undergo before becoming functional, translatable mRNAs in the cytoplasm.

Proteins that decreased binding to the R2 by TNFα

Protein	Functions
FBRL	S-adenosyl-L-methionine-dependent methyltransferase that has the ability to methylate both RNAs and proteins. Site specificity is provided by a guide RNA that base pairs with the substrate. Methylation occurs at a characteristic distance from the sequence involved in base pairing with the guide RNA.
BAZ1B	Atypical tyrosine-protein kinase that plays a central role in chromatin remodeling and acts as a transcription regulator. Essential component of the WICH complex, a chromatin remodeling complex that mobilizes nucleosomes and reconfigures irregular chromatin to a regular nucleosomal array structure.
RBP2	E3 SUMO-protein ligase which facilitates SUMO1 and SUMO2 conjugation by UBE2I. Involved in transport factor (Ran-GTP, karyopherin)-mediated protein import via the F-G repeat-containing domain which acts as a docking site for substrates. Binds single- stranded RNA (in vitro). May bind DNA.
PRP8	Functions as a scaffold that mediates the ordered assembly of spliceosomal proteins and snRNAs.
NOP58	Required for 60S ribosomal subunit biogenesis.
DDX41	Probable ATP-dependent RNA helicase. Is required during post-transcriptional gene expression.
DDX52	Probable ATP-dependent RNA helicase.
U520	RNA helicase that plays an essential role in pre-mRNA splicing as component of the U5 snRNP and U4/U6-U5 tri-snRNP complexes.

Supplementary Table 1. List of identified molecules whose affinity to the R2 was changed by inflammatory signals and summary of each function of them.

Primer sequences used for bisulfite sequencing

Gene	Sequence (5' to 3')	Direction
Mouse Adiponectin R1	AGGTAAGTGTTTTGTGATATTGGGT	Forward
	ACACCCACAATAATTCCATAAAATC	Reverse
Mouse Adiponectin R2	TGGAGGAAGTAGATGTTTGGTTAGT	Forward
	CAAAACAATACCTTAAAAACCTCTC	Reverse
Human Adiponectin R2	GGGGTAGGTAGATATTTGTTTTGTTT	Forward
	TCAACACCTTAAACTTTCTTAACA	Reverse
Mouse PPARγ2	GATGTGTGATTAGGAGTTTTAATTAAAG	Forward
	CAAACCTAAATTAACTAACACTATCCTAAC	Reverse
Mouse TNFα	TAGATTGTTATAGAATTTTGGTGGG	Forward
	TTCTATTCTCCCTCCTAACTAATCC	Reverse
Mouse MCP-1	TGGTAATTATTAAGTGGAGAGAATGTT	Forward
	TTAATACCAAAAAATAACATCACCCTAA	Reverse

Primer sequences used for restriction enzyme accessibility assay and ChIP

Gene	Sequence (5' to 3')	Direction
Mouse Adiponectin R2	CTTTGCCTGGGAGCAGTCTA	Forward
	CTTAAAAGGCTTGCAGTTGG	Reverse

Supplementary Table 2. Primer sequences used for bisulfite sequencing, restriction enzyme accessibility assay, and ChIP.

Gene	Sequence (5' to 3')	Direction
Mouse and human *adiponectin*	GGCAGGAAAGGAGAACCTGG<br>AGCCTTGTCCTTCTTGAAGAG	Forward<br>Reverse
Mouse *Dnmt1*	CGGCTCAAAGACTTGGAAAG<br>TAGCCAGGTAGCCTTCCTCA	Forward<br>Reverse
Human *DNMT1*	GAGGAAGCTGCTAAGGACTAGTTC<br>ACTCCACAATTTGATCACTAAATC	Forward<br>Reverse
Mouse *Dnmt3a*	CGACCCATGCCAAGACTCACCTTCCAG<br>AGACTCTCCAGAGGCCTGGT	Forward<br>Reverse
Mouse *Pparγ2*	GCATGGTGCCTTCGCTGA<br>TGGCATCTCTGTGTCAACCATG	Forward<br>Reverse
Mouse *C/ebpα*	CAAGAACAGCAACGAGTACCG<br>GTCACTGGTCAACTCCAGCAC	Forward<br>Reverse
Mouse *Tnfα*	CGGAGTCCGGGCAGGT<br>GCTGGGTAGAGAATGGATGAACA	Forward<br>Reverse
Mouse *Mcp-1*	AGGTCCCTGTCATGCTTCTG<br>TCTGGACCCATTCCTTCTTG	Forward<br>Reverse
Mouse *Il-1β*	TGCAGAGTTCCCCAACTGGTACATC<br>GTGCTGCCTAATGTCCCCTTGAATC	Forward<br>Reverse
Mouse *inos*	AATCTTGGAGCGAGTTGTGG<br>CAGGAAGTAGGTGAGGGCTTG	Forward<br>Reverse
Mouse *Il-6*	CTTCCATCCAGTTGCCTTCTTG<br>AATTAAGCCTCCGACTTGTGAAG	Forward<br>Reverse
Mouse *Grp78*	ACTTGGGGACCACCTATTCC<br>TTTCTTCTGGGGCAAATGTC	Forward<br>Reverse
Mouse *Chop*	GTCCCTAGCTTGGCTGACAGA<br>TGGAGAGCGAGGGCTTTG	Forward<br>Reverse
Mouse *Vegfα*	GGAGATCCTTCGAGGAGCACTT<br>GGCGATTTAGCAGCAGATATAAGAA	Forward<br>Reverse
Mouse *Glut1*	ACTGGGCAAGTCCTTTGAGA<br>GTCTAAGCCAAACACCTGGGC	Forward<br>Reverse
Mouse *Srebp1c*	GGAGCCATGGATTGCACATT<br>CAGGAAGGCTTCCAGAGAGG	Forward<br>Reverse
Mouse *Fas*	GCCTACACCCAGAGCTACCG<br>GCCATGGTACTTGGCCTTG	Forward<br>Reverse
Mouse *Scd1*	CCGGAGACCCCTTAGATCGA<br>TAGCCTGTAAAAGATTTCTGCAAACC	Forward<br>Reverse
Mouse *cyclophilin*	CAGACGCCACTGTCGCTTT<br>TGTCTTTGGAACTTTGTCTGCAA	Forward<br>Reverse
Human *GAPDH*	TGCACCACCAACTGCTTAG<br>GGATGCAGGGATGATGTTC	Forward<br>Reverse

Supplementary Table 3. Primer sequences used for quantitative real-time PCR
